# Supplementary material for: Content-rich biological network constructed by mining PubMed abstracts
Source: BMC Bioinformatics. 2004 Oct 8;5:147. doi: 10.1186/1471-2105-5-147 (PMC528731; doi:10.1186/1471-2105-5-147)
Supplement: Additional File 5 — The original Chilibot query results of the term "long-term potentiation (LTP)" and 22 other terms, limiting the latest references analyzed to the years 1990, 1995, 2000, and 2004. [file 1471-2105-5-147-S5.bz2 › chilibotAdditionalFile5/ltp2000/html/PI-3K_PKA.html]

 


 **PI-3K** and **PKA** 
  
Found 21 abstracts in PubMed,  **21 abstracts were retrieved and analyzed**.  


---

 Search Google  |
 PDF files only 
|  EDU domain only 

---

**Interactive relationship** (e.g. stimulation, inhibition, etc)

- Together, these results show that cross talk between the mu opioid receptor and the MAP kinase cascade is not mediated by the cAMP  **PKA**  pathway, but involves PI 3 kinase  [ **PI-3K** ] .  Ref: 10452557 FEBS Lett, 1999

**Parallel relationship** (e.g. studied together, co-existance, homology, etc.)

- Inhibitors of the beta isoform of PKC LY333531, protein kinase A  **PKA**  H89, and phosphotidylinositol PI 3 kinase  [ **PI-3K** ]  wortmannin had no significant effect.  Ref: 10331422 Diabetes, 1999
- It also inhibits other downstream serine threonine kinases, such as  **PKA**  and p90 S6 kinase, but not upstream tyrosine phosphorylation or PI3 kinase  [ **PI-3K** ]  activation in response to insulin.  Ref: 10679201 Biochem Biophys Res Commun, 2000
- Thus, insulin inhibition of  **PKA**  induced PEPCK expression does not require MAPK activation but does require activation of PI3 kinase  [ **PI-3K** ] , although this signal is not transmitted through the PKB or PKC pathways.  Ref: 9668048 J Biol Chem, 1998
- The effect of ET 1 on glucose uptake was not inhibited by inhibitors for protein kinase C PKC, protein kinase A  **PKA**  and phosphatidylinositol 3 kinase PI3 kinase  [ **PI-3K** ] .  Ref: 11078371 J Cardiovasc Pharmacol, 2000
- We suggest that Bu 2cAMP stimulates Glut 1 translocation to plasma membrane through PI3 kinase  [ **PI-3K** ]  dependent and  **PKA**  independent signaling pathways.  Ref: 11089547 Endocrinology, 2000
- PI 3 kinase  [ **PI-3K** ]  inhibitor wortmannin, PKC inhibitor H 7, or  **PKA**  inhibitor HA1004 did not suppress adhesion induced by IL 3 or Flt3 ligand.  Ref: 9682000 Cell Immunol, 1998
- Likewise, Bu 2cAMP effects on glucose transport and Glut 1 translocation to plasma membrane were repressed by PI3 kinase  [ **PI-3K** ]  inhibitors but not by the protein kinase A  **PKA**  inhibitor H89.  Ref: 11089547 Endocrinology, 2000
- Somatostatin exerts its antiproliferative effect inhibiting more upstream the TSH stimulation of  **PKA**  and PI 3 kinase  [ **PI-3K** ] , interfering with the TSH mediated increases of intracellular cAMP levels by inactivation of adenylyl cyclase activity.  Ref: 10809788 J Biol Chem, 2000
- In this report, we show that PKB can also be activated by  **PKA**  cyclic AMP cAMP dependent protein kinase through a PI3 kinase  [ **PI-3K** ]  independent pathway.  Ref: 10373549 Mol Cell Biol, 1999
- These data suggest that bFGF stimulates KDR expression through a PKC and p44 p42 MAPK dependent pathway not primarily involving the beta isoform of PKC,  **PKA** , or PI 3 kinase  [ **PI-3K** ] .  Ref: 10331422 Diabetes, 1999
- in contrast, PLC inhibitor U 73122 did suppress adhesion, suggesting the possibility that PLC... PKC, or  **PKA** , may be involved in this process.  Ref: 9682000 Cell Immunol, 1998
- Together, these results suggest the existence of a  **PKA**  dependent pathway and a new  **PKA**  independent PI 3 kinase  [ **PI-3K** ]  pathway in the TSH cAMP mediated proliferation of FRTL 5 thyroid cells.  Ref: 10809788 J Biol Chem, 2000
